# Competitor-Weighted Centrality and Small-World Clusters in Competition Networks on Firms’ Innovation Ambidexterity: Evidence from the Wind Energy Industry

**DOI:** 10.3390/ijerph20043339

**Published:** 2023-02-14

**Authors:** Runbo Zhao, Huiying Zhang, Marina Yue Zhang, Fei Qu, Yunlong Xu

**Affiliations:** 1 College of Management and Economics, Tianjin University, Tianjin 300072, China; 2 Australia-China Relations Institute, University of Technology Sydney, Sydney 2007, Australia; 3 Business School, Guilin University of Technology, Guilin 541004, China; 4 School of Civil Engineering, Tianjin University, Tianjin 300072, China

**Keywords:** structure embedding, technology competition network, green technology innovation, ambidexterity, wind energy, innovation performance

## Abstract

A firm’s embedding structures in a technology competition network can influence its propensity for innovation ambidexterity. Using PCT (patent cooperation treaty) patent data of wind energy companies between 2010 and 2019, we adopted social network analysis and fixed-effects panel negative binomial regression to examine the impacts of network structural features on firm innovation ambidexterity. The results show that competitor-weighted centrality contributes to a firm’s propensities for both incremental and radical green innovation. In contrast, a firm’s embeddedness in small-world clusters can moderate the effect of the firm’s competitor-weighted centrality positively on its incremental innovation but negatively on its radical innovation. The study makes three theoretical contributions. First, it enriches the understanding of how the competition network affects innovation ambidexterity. Second, it provides new insights into the relationship between competition network structures and technology innovation strategy. Finally, it contributes to bridging the research on the social embeddedness perspective and green innovation literature. The findings of this study have important implications for enterprises in the wind energy sector regarding how competitive relationships affect green technology innovation. The study underscores the importance of considering the competitiveness of a firm’s rivals and the embedded structural features when devising green innovation strategies.

## 1. Introduction

Severe environmental challenges and energy shortages have aroused widespread concerns and led to the burgeoning of studies on green or sustainable innovation in recent years (e.g., [1,2,3]). Green innovation, also called sustainable innovation, is innovation applied in environmental practices, energy conservation, waste reduction, pollution prevention, etc. [4]. As a critical type of green innovation, green technology innovation can help reduce the environmental burden and drive the technological upgrading of the economy [5,6] and has become the strategic focus of enterprises. The concept of ambidexterity finds a wide range of applications across various organizational contexts. There is a growing consensus that organizational ambidexterity indicates companies’ ability to simultaneously explore and exploit [7]. In this paper, the ambidexterity of green technology innovation is defined as two essential enterprise strategies: Incremental green technology innovation refers to the continuous improvement that leverages existing technology, while radical green technology innovation indicates that significant progress deviates from current technology. Enterprises gain sustainability competitiveness when they constantly engage in both exploratory and exploitative activities [8]. That is, firms must not only carefully consider the pressure on current technology regimes but also capture opportunities for new technologies [9].

How competition network structure affects firm performance has attracted considerable attention in recent years [10]. Compared with the cooperation network (e.g., [11,12,13]), information flow in a firm’s competition network occurs at a lower frequency [14], and the information is more public rather than private. Firms cannot ask for information directly from their rivals, which urges firms to spend more effort to search for and decode information on competitors’ dynamics [15], especially in more tacit technology competition networks [16]. Therefore, a favorable position in a competition network is especially crucial for a firm’s innovation activities. In the study of how competitive network structure affects firm performance, relevant findings include that the size of a competition network can increase the firm’s product market entry [10], the competitive density and strength affect the firm’s technology competitive capability differently [16], inter-organizational linkages reduce the likelihood of competitive war [17], and the position of brokerage in the domestic technology competition network can facilitate the firm to join related international strategic alliances [15]. Although these studies have extended our understanding of the competition network structure, several essential research gaps remain to be explored further:

(1) Many studies treat competitors in a competition network equally by measuring the intensity of competition by the number of competitors. We argue that the different competitive ability of the competitors deserves academic attention. In practice, one strong competitor plays a completely different role for a local firm than many ordinary competitors combined.
(2) Previous studies rarely distinguish how structural features embedded in a competition network affect the two modes of firm-level green technology innovation, exploration vs. exploitation.

To fill these gaps, this paper examines the effect of two network structures on the ambidexterity of green technical innovation from the perspective of social embeddedness. The first structural feature is competitor-weighted centrality. (1) Centrality takes into account that nodes in different network locations have different levels of importance. A firm in a more central position can gather more information and knowledge from the network [18], which helps monitor its competitors’ dynamics, reduce the uncertainty of technological direction, and seize potential opportunities for technical development [10,14,19]. (2) Competition weighting includes the centrality of competitors; that is, more weight is assigned to a focal firm’s centrality if its key rival is central in the competition network. However, enterprise nodes are also affected by neighboring enterprises [20]. When facing a strong competitor, the enterprise will have greater competitive pressure, prompting it to keep searching for and learning from the network [14] and improving the firm’s capabilities. Thus, focusing on a firm alone without considering its rival’s competitiveness can result in a cognitive bias in understanding the strategic choice of the focal firm in technology innovation. Based on the measurement method of Qi’s research (2016) in cooperative networks, this paper innovatively constructs the competitor-weighted centrality, which considers both the features of both a focal firm and its competitors, measuring the firms’ technical competition in the competitive network more accurately.

The second structural feature is a firm’s embeddedness in small-world clusters. This paper focuses on its moderator role in the firm’s green technology innovation. Firms in small-world clusters tend to have more tensive connections with each other but relatively sparse ties with firms outside the clusters [21,22]. The tensive connections in a competition network and repeated interactions can reduce the diversity of competitors [10], make the connections more interdependent [23], and thus, promote mutual trust that aligns with the rivals’ interests [24]. As a result, the more a firm is embedded with specific competitors, the more sensitive it is to changes in the rival dynamics and the quicker it can respond to the changes by conducting incremental innovation. However, the capture of homogeneous information from tight connections can spur only conventional reactions [25] on existing paths [26]. Only when firms are stimulated by new external factors will they begin to expand their innovation directions [27,28]. Furthermore, being embedded in different external environments brings firms different technical pressures, motivating them to search for new directions [29]. From this stream of argument, we deduce that embeddedness in small-world clusters can have differential impacts on the effect of the competition network structure on ambidextrous strategies for green technical innovation.

Wind energy is chosen as the empirical setting for the following reasons. In the context of a low-carbon economy, wind energy plays a vital role in reducing carbon emissions to mitigate climate change [30]. Moreover, it is the most rapidly growing and the most promising renewable energy source [30,31], which has drawn great attention from governments and companies in recent years. For example, the Chinese government has promulgated a series of subsidies and tax credits to support companies involved in wind technology [16,32]. These initiatives have greatly contributed to sustainable economic development and ensured energy security. For enterprises with increasing complexity and growing demand for advanced wind energy products, there is a constant push to innovate their technologies, which enables them to keep technological advantages in the competitive market. All of these reasons made technological innovation by wind energy enterprises an important issue worthy of further examination.

In summary, we combine the social embeddedness perspective with studying how the two structures of the competition network (the competitor-weighted centrality and embedding in small-world clusters) affect the ambidexterity of a firm’s green innovations. We test the propositions in the wind energy field, which is deemed a promising source of clean energy [31], using patent data from companies between 2010 and 2019. Our contributions are as follows: (1) This study enhances our understanding of how competitive structures influence the ambidexterity of a firm’s green technical innovation, including radical and incremental innovation. (2) We reveal how the weighted centrality of the technical competition network impacts a firm’s innovation by counting the centrality of the focal firm’s competitors. (3) We extend the social embeddedness perspective by illuminating the moderator role of embedding in small-world clusters. The small world is a suitable embodiment for understanding the competitive environment in which a firm is embedded. (4) We propose several management practices for managers of wind energy enterprises facing a competitive environment for technology.

## 2. Theoretical Foundation

### 2.1. Wind Energy Studies

According to the World Energy Outlook 2021 report, annual clean energy investments will grow to USD 4 trillion by 2030 to achieve the goal of net zero emission of CO_2_ [33]. Among renewable energy sources, wind energy contributed 34% of the newly installed renewable energy capacity in 2016 [34]. In 2020, wind energy even increased at the fastest rate under the context of economic downturns during COVID-19 lockdowns [33], which shows a huge potential for wind power.

Previous studies have made great contributions by analyzing the wind energy industry from different aspects (e.g., policy, institutional logic, product design, cooperation network, etc.). Shen (2019) investigated how government regulation affected stakeholders. They uncovered that the delegation of approval authority promotes the growth of regional wind power and suggest that governments carefully consider the trade-off between different levels of approval authority [32]. Yock (2016) conducted research based on the conflict argument on wind energy (i.e., natural environment in conflict with economic prosperity) from the perspective of institutional logic and the evolution of organizational fields [30]. Yang (2021) proposed a framework to improve the success rate of the radical innovation of wind power systems. They used the QFD (quality function deployment) method to discover features of radical technologies and the TRIZ (theory of innovative problem solving) method to instruct the design process of wind energy products [35]. Liu (2021) studied the cooperation network of the wind energy industry through patent analysis. They suggested Chinese government strengthen the construction of cooperation platforms and encourage technological innovation [13].

Although these studies have made great contributions to the wind energy industry, little attention has been paid to the perspective of innovation strategy. Our research, therefore, investigates how the embedding structures of technology competition networks affect firm performance in innovation in the wind energy sector.

### 2.2. The Ambidexterity of Green Technology Innovation

The ambidexterity of exploration and exploitation in green technology innovation is a vital strategy for firm performance: incremental innovation, based on exploiting existing technologies, contributes to firms’ current growth; while radical innovation, based on exploring breakthrough technologies, facilitates firms’ long-term performance [36]. As an enterprise’s resources are limited, a trade-off between these two types of innovation strategies needs to be carefully considered for firms to optimize their performance.

We follow the classification of green innovation in Cui’s (2022) research and divide green technology innovation into two types: incremental and radical green technology innovation [1]. Incremental green technology innovation refers to green technology development with continuous changes based on exploiting existing technology. Radical green technology innovation indicates that technological advancements have significantly progressed by exploring new technologies away from the present ones. These two types of green technology innovation are essential for enterprises’ growth. On the one hand, radical innovation can lead to valuable technical and financial improvement [37], and it also provides enterprises with considerable technical advantages, innovation competitiveness, and reputation [38]; however, it also carries intrinsic uncertainties and risks (March, 1991). On the other hand, incremental innovation is more likely to succeed [39,40], but it risks missing breakthroughs in the industry and being interrupted by newcomers (Christensen, 1997).

Patent data is an important indicator of technological innovation and is widely used to measure the technological innovativeness of firms and nations (e.g., [41,42]). Compared with other intellectual properties, patents are transferable assets with both economic and technological value, making patents better reflections of the reality of technological development [43]. We collected the green patents according to the IPC (International Patent Classification) Green Inventory, developed by experts in the World Intellectual Property Office, to reflect enterprise green technology innovation activities. In this paper, green patents are divided into two categories according to their novelty. Radical green patents are patents that contain new green technological sub-classes, and incremental green patents are patents that contain only existing green technological sub-classes [41]. The detailed measurement is introduced in Part 4.3.1. Based on these green patents, we can understand the input and output of enterprises in green technology research and development, competitive relations, and other information, all of which help provide useful suggestions to improve enterprises’ green technology innovation and improve energy efficiency.

### 2.3. Antecedents of Firms’ Green Technology Innovation

Previous studies have investigated various antecedents of a firm’s green technology innovation, including the following aspects: (1) External factors that mainly influence the policy environment. Scholars have explored the effect of carbon emission trading policy on a firm’s technology innovation [3], the impact of environmental regulation on technological innovation efficiency [44], how different political competition influences the enterprise’s green technology innovation [45], the effect of China’s R&D investment on green innovation performance [46], and how the green credit policy impacts a firm’s green technology innovation [47]. (2) Internal factors that mainly influence the organizational learning process. The research includes, for example, how firms change their open innovation strategies to develop green competence-destroying technologies [48], and the exploration process of green technology innovation from a learning perspective is based on a case study [49]. These studies have contributed significantly to the green technology innovation literature. In addition, a firm’s performance can also be affected by its surrounding enterprises [20]. A unique position in a technology competition network can be a lever that creates useful differentiation for a focal firm, particularly regarding its innovation, and may help explain why seemingly similar firms differ in their innovation outcomes. Therefore, how structural embedding features influence a firm’s innovation strategy in a technology network merits more academic investigation.

### 2.4. Definition of Two Structural Features

**Figure 1:**
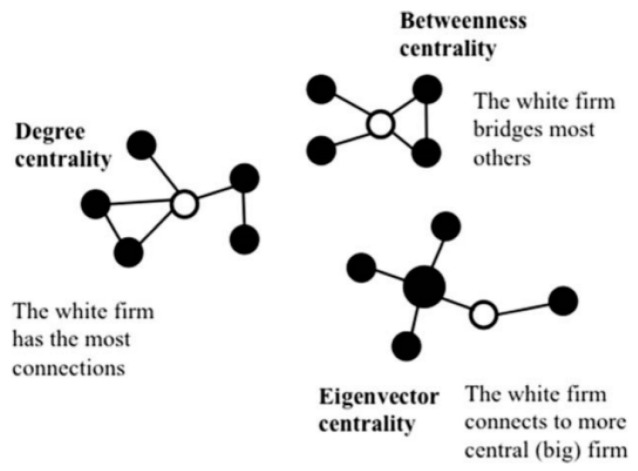
Three common forms of network centrality (Reprinted with permission from Ref. [11]. 2017, John Wiley & Sons—Books).

Competitor-weighted centrality refers to the degree to which a focal enterprise competes with other firms in a technology competition network. This feature considers not only the number of competitors but also the quality of the competitors in the network. That is, the focal firm with more central competitors in the competition network is given more ‘weight’, and a firm with more peripheral rivals is given less ‘weight’, when calculating its value of competitor-weighted centrality. This is because the more central a firm’s position is in the network the more easily it can attract resources in the network [50,51]. Figure 1 illustrates three common forms of network centrality. Unlike degree centrality and betweenness centrality, which treat other nodes as having equal importance, eigenvector centrality is more sophisticated [11], as it can capture the heterogeneities of other nodes. Therefore, we employ eigenvector centrality to measure the ‘competitor-weighted centrality’ of focal firms in this paper. Given that competitor-weighted centrality measures not only how many competitors the focal firm competes with but also the difference in the competitiveness of the competitors, we believe this measure is closer to the real-life situation of competition.

**Figure 2:**
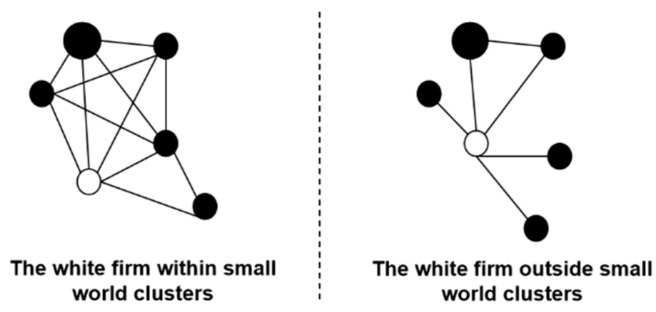
The embeddedness within small-world clusters.

Small-world clusters can reflect the external competitive environment in which an enterprise is embedded. Figure 2 shows the distinguishing features of whether an enterprise is embedded in small-world clusters. Firms embedded in small-world clusters tend to build more connections with each other, while they have few connections outside of the clusters [12,21,22]. Since the environment in which a firm is embedded has a significant influence on its competitive capacity [16] and search strategies [52], exploring the differences in enterprises’ embeddedness in small-world clusters may help explain why similar firms, at face value, differ in their innovation performance.

## 3. Hypotheses

This section analyzes the effects of the two significant embeddedness structures of a competition network. Hypotheses 1a and 1b examine the main effect of competitor-weighted centrality on network firms’ radical and incremental green innovation, respectively. Hypotheses 2a and 2b investigate the moderator effect of embeddedness in small-world clusters on network firms’ ambidexterity of green technology innovation, respectively.

### 3.1. The Main Effect of Competitor-Weighted Centrality

First, enterprises with higher competitor-weighted centrality suffer from higher competitive pressure, which is one of the strongest drivers of corporate innovation. Generally, if a firm has higher centrality in a technology competition network, it means that it has formed a competitive relationship with many enterprises in the network and can, therefore, receive more competitive pressure. This type of pressure from the competitors can stimulate a focal firm to search for and learn from the competitors [14], which can be translated into the firm’s technology strategy [53]. Hence, we argue that competitor-weighted centrality can better capture the pressure of competitive relations surrounding the focal firm and will benefit their green innovations.

Second, competitor-weighted centrality can bring diverse and timely information to the firms within a network, which is a valuable resource for firms’ innovation. The competitive relationships within a competition network can be an important channel for information flows among rivals. Since information can be conveyed through the competitors’ actions [54], enterprises can, thus, gather information by monitoring the dynamics of their competitors. A high frequency of interaction with various competitors can help capture the timing and diverse information, which can benefit the central firms in the network [15]. This kind of information can be helpful in competitive analysis and strategical formulation [55], and it can bring new opportunities to firms occupying such positions [54,56]. Furthermore, information is crucial to innovation, as it helps reduce the uncertainty of technological competition; prepare, in advance, for potential technological threats; and predict the future directions of technology development [14]. In addition, if a focal firm is more likely to acquire technical information that others cannot access, it will obtain the first-mover advantage, leaving its rivals in a position that is hard to catch up from in a short time [14].

Altogether, competitor-weighted centrality enables focal firms to perceive higher competitive pressure, which can motivate them to actively engage in searching and learning activities and, hence, obtain useful information. As a result, such centrality can lead to both exploratory and exploitative innovations in green technology. Hence, we posited the following hypotheses between a firm’s competitor-weighted centrality in a competition network and its green technology innovation.

**H1a:**  *A firm’s competitor-weighted centrality positively affects its radical green technology innovation in a competition network.*

**H1b:**  *A firm’s competitor-weighted centrality positively affects its incremental green technology innovation in a competition network.*

### 3.2. Moderating Effect of Small-World Clusters

First, firms can experience different competitive pressures when they are inside and/or outside small-world clusters. The pressure that a firm experiences within the same small world tends to be homogeneous, while the pressure is heterogeneous when they are not in the same small world. Different types of competitive pressure can lead to different motivations for innovation [29]. This is because a dense competition network can reduce the diversity of competitors [10] and enable the competitors to act more interdependently and in a similar fashion [23]. Repeated interactions can facilitate knowledge exchange and resource sharing [17], leading to the promotion of mutual trust and alignment of interests among competitors [24,57]. Consequently, focal firms in small-world clusters are more sensitive to their competitors’ technological dynamics and act more rapidly in response to others’ actions at a lower cost. Hence, embeddedness in small-world clusters can lead focal firms to have a higher tendency toward incremental innovation. However, a homogenous technological foundation can deter the firms’ radical innovation. Potential opportunities, new trends, and radical innovation, thus, become difficult if firms are embedded too deeply in specific clusters [24]. By contrast, firms outside of small-world clusters face competitors from more diverse backgrounds. The surrounding environment makes it impossible for such firms to respond as quickly as the ones inside small-world clusters, and, thus, drives firms outside of the small-world cluster to explore new innovation directions that match their environment.

Second, firms embedded in small-world clusters obtain different types of information from those outside the clusters, which can instigate different reactions (search orientation) within firms. Since information within a small world is shared in an intensified information flow [58], firms inside the small-world cluster are more likely to capture familiar information on a common technical foundation. When capturing conventional information, firms tend to respond in a conventional way and engage in less exploratory innovation [25], whereas novel or unfamiliar information has the ability to attract focal firms’ attention and encourage their search for solutions away from their technological trajectories [14]. The unknown result brought by novel or unfamiliar information can spur such firms to engage in outward searching and exploratory innovation. We argue that embeddedness in a small world contributes to a firm’s incremental innovation but not to its radical innovation. Over the long term, internal embeddedness can reinforce firms’ cognitive barriers and lock them in knowledge isolation, resulting in a narrower scope of innovation [59]. On the contrary, information with connections to outside firms can stimulate firms to search for novel technologies [22,60], and technical knowledge from distant fields can encourage creativity and foster new ideas that are more likely to become radical innovations [25,61,62].

Based on the above arguments, enterprises embedded within or outside of small-world clusters tend to experience different competitive pressures and receive different information, which spurs their different responses and leads to them adopting innovation strategies. Hence, we posited the following hypothesis:

**H2a:**  *Embeddedness in small-world clusters negatively moderates a firm’s relationship between its competitor-weighted centrality and radical green technology innovation.*

**H2b:**  *Embeddedness in small-world clusters positively moderates a firm’s relationship between its competitor-weighted centrality and incremental green technology innovation.*

**Figure 3:**
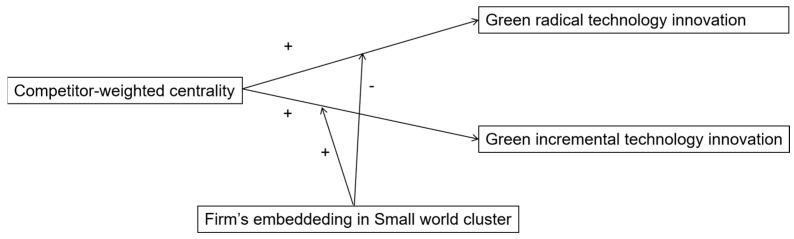
Research framework. “+” indicates the positive effect; “−” indicates the negative effect.

Based on the preceding discussion, our research framework is shown in Figure 3.

## 4. Methodology

### 4.1. Data and Collection

The research panel data mainly included patent data and enterprise attribute information. First, we followed a series of steps to ensure the accuracy of the patent data. (1) The formulation of a search string. Our collection of wind energy green patents was built on the integrated use of keywords and green technical categories. We referred to previous studies [31,63], to obtain the relevant keywords for wind energy. We learned of the green technical category of F03D for wind energy from the IPC Green Inventory, developed by experts in the World Intellectual Property Office (https://www.wipo.int/classifications/ipc/green-inventory/home (accessed on 1 January 2023)). (2) The choice of patent application type. Since a patent application is a perfect agent for a firm’s innovation output [41], we employed application data from the United States Patent Office (USPTO) during 2010–2019. The reason for choosing patents applied for within the USPTO was because it is a good representation of a firm’s global innovation performance [64], and USPTO contains plenty of high-quality patents within the largest renewable energy market of wind energy. (3) The selection of the patent period. The review of patent applications sometimes takes two or three years, depending on the complexity of the patent text and other factors. It was likely that not all patents applied for after 2020 would have completed their examination by 30 June 2022 (the date we collected these data). This prompted us to choose the years 2010–2019 as our observation period and not include the very recent years. In this way, we could ensure that all patents applied for during this period were collected, and thus, reflected the technical innovation performance of the company in the best possible way.

Altogether, our search string was CTB = (“wind power” OR “wind energy” OR “wind turbine” OR “wind generator” OR “wind electricity” OR “wind farm” OR “windmill” OR “energy of wind” OR “energy from wind” OR “wind rotor” OR “wind axis” OR “wind blade”) AND ICR = (F03D) AND AY ≥ (2010) AND AY ≤ (2019) AND AC = (US). We conducted data collection on 30 June 2022, within the Derwent Innovation (DI) database—the largest commercial patent database in the world, composed of many authoritative organizations worldwide [65]. Eventually, 5034 patents were obtained.

Second, we collected enterprise attribute information. Since the 5034 collected patents covered more than 1000 enterprises, we found that the number of patents owned by the top 120 enterprises was 3752, accounting for about 74.5% of the total. According to the Pareto principle, the top enterprises create the vast majority of patent resources. We finally collected the attribute information of the top 120 representative enterprises. The attribute information, including the number of employees, firm age, and cash flow, was then collected from the Compustat database within Wharton Research Data Services (WRDS), the EBSCO database, and some corporate annual reports.

### 4.2. Construction of Firm’s Technical Competition Network 

**Figure 4:**
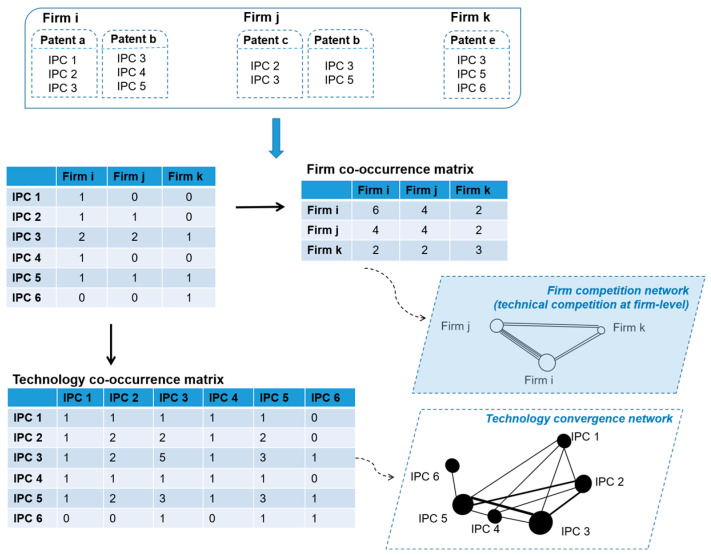
Construction of a firm’s competition network.

Figure 4 shows the construction process of the firms’ green technology competitive network. First, the IPC codes, representing the wind energy technologies in a firm’s patent, were extracted. Second, enterprises with the same IPC codes indicated that they had technically competitive relationships. Thus, the greater the number of common IPC codes, the more intense their technical competition was. Third, according to the competitive relationship obtained in the previous step, we summarized their competitive relationship within the firm co-occurrence matrix. Finally, the firm competition network was constructed based on the firm co-occurrence matrix.

Further, although the firm competition network is the main focus of this paper, we would like to briefly mention another interesting idea: the technology convergence network. As Figure 4 illustrates, we also constructed a technology convergence network. Different firms with the same IPC code tended to have a competitive relationship; however, at the technology level, the higher frequency of different IPC codes within the same enterprise indicated a closer relationship between these IPC codes. That is, there was a higher convergent tendency for these different technologies. Similarly, a technology convergence network could be constructed based on the technology co-occurrence matrix [66]. We speculate that there is a specific relationship between technology convergence and firm competition networks, and their interaction will be an interesting topic in future research to provide insights into firms’ technical competition activities.

**Figure 5:**
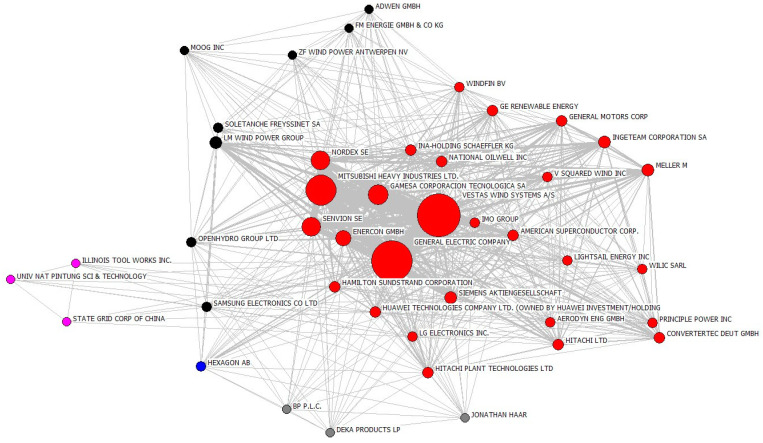
Firm competition network in 2010.

Figure 5 shows an example of a competition network of wind energy companies in 2010. Each node within the network is a firm with wind energy patents. The size of a node represents its competition-weighted centrality. The link between them indicates that they had common IPC codes. Therefore, the thicker the link, the more overlapping the two firms’ technologies areas were, and the stronger their competitive relationship. 

### 4.3. Measurement

#### 4.3.1. Dependent Variables

Based on existing literature on the measurement of exploration and exploitation innovation [41], this paper measured the ambidexterity of green technical innovation in the following ways. Green technical radical innovation indicated the degree of that firm’s utilization of new green technology, which was measured by the total number of patents containing new green technological classes. A new technological class referred to a technical class that a firm had not used in patents filed within the previous five years. The five-year window is appropriate for estimating innovation and is widely used [64,65,67,68]. Therefore, a patent that utilized a new technological class was categorized as a technical exploration outcome. Similarly, green technical incremental innovation captured the firm’s reuse of existing green technologies, which were measured by the total number of utilizations of familiar technical classes (which had appeared at least once) within the previous five years. Patents that used only these familiar technical classes were considered incremental outcomes.

#### 4.3.2. Independent Variables

We measured the competitor-weighted centrality by the eigenvector centrality degree. As stated earlier, eigenvector centrality is a more advanced index that can capture the differences between adjacent nodes. If its adjacent nodes also have higher centrality, then the focal node will have more influential power and capability than the others [11,69,70]. The formula is as follows:

(1) $$\mathrm{ei}g({firm}_{i})=\frac{1}{\lambda }\sum_{k\in M}\mathrm{ei}g({firm}_{k})$$

Among these, $\mathrm{ei}g({firm}_{i})$ indicates the eigenvector centrality of ${firm}_{i}$, $\frac{1}{\lambda }$ indicates the eigenvalue, and *k* indicates the direct competitor of firm *i* (i.e., having a direct competition with the firm *i*). *M* is the set of all direct competitors of firm *i*. These parameters and each node’s eigenvector centrality were calculated using the Ucinet 6 tool.

#### 4.3.3. Moderator 

The average clustering coefficient is widely used to measure the degree of node clusters with neighboring nodes within a closely related group [12,71,72]. A higher value meant that a firm had dense connections with neighboring knowledge within a small-world cluster, and all connections in the network were scattered in the distribution [12,72]. The formula for the average clustering coefficient is as follows:

(2) $${ACC}_{i}=\frac{n_{k}}{n_{i}(n_{i}-1)/2}$$

${ACC}_{i}$ represents the average clustering coefficient of firm *i*; that is, the small-world value of firm *i*. $n_{k}$ stands for the number of connections among all $n_{i}$ direct competitors, *k*, of the focal firm, *i*.

#### 4.3.4. Control Variables

To avoid the endogeneity problem, we selected control variables based on firms, unlike the dependent and independent variables built on patents. The detailed control variables included the firm’s size, cash flow from operating activities (CFOA), R&D expenditures, age, and technology elements. All these variables impacted the firm’s technology innovation activities, and their respective measurements, references, and data sources are represented in Table 1.

### 4.4. Analysis Strategy

Fixed-effects models can help control for time-invariant factors [14]. Moreover, since our dependent variables were non-negative integer values with a skewed distribution, linear regression models could lead to inconsistent, biased, and inefficient estimates [77]. Therefore, the fixed-effects panel negative binomial model was the most appropriate for this study. Further, we clustered by firm and used robust standard errors in all analyses to correct the correlation of observations from the same firm over the year.

## 5. Results

### 5.1. Descriptive Statistical Analysis

This section illustrates the results of our procedure. Table 2 reports the descriptive statistics and correlations. The variance inflation factors (VIFs) for all the variables were below the recommended cut-off of 5.0, indicating that collinearity was unlikely to affect our results. Generally, firms produce an average of 2.7 radical and 3.7 incremental patents, yearly. In addition, competitor-weighted centrality has a positive effect on both the firm’s radical and incremental innovation (β_1_ = 0.917, *p*_1_ < 0.01; β_2_ = 0.956, *p*_2_ < 0.01). Notably, we observed that the Smallworld variable negatively affected radical innovation to a greater extent than incremental innovation (β_1_ = −0.259, *p*_1_ < 0.01; β_2_ = −0.213, *p*_2_ < 0.01), which indicates that a firm’s truck in a small world is not beneficial to their innovation outcome, especially the radical outcome. The impact of the interaction between the small world and competitor-weighted centrality on innovation will be discussed in the next section. 

### 5.2. Regression Analysis

We first ran the fixed-effects negative binomial panel regression, and the results of the analyses are presented in Table 3. Models 1.1 to 1.4 provide the results of radical green innovation, and Models 2.1 to 2.4 show the results of incremental green innovation.

Hypotheses 1a and 1b predicted that competitor-weighted centrality would be positively related to radical and incremental green innovation, respectively. Their coefficients in the results were significant and positive across the models, supporting Hypotheses 1a and 1b.

Hypothesis 2a predicted that being embedded in a small world would weaken the effect of competitor-weighted centrality on a firm’s radical innovation. We observed that the coefficient of Smallworld (β = −0.219, *p* < 0.01) was negative and significant (Model 1.3), which means that enterprises being structured in the small world was not beneficial for their radical innovation. In a further step, we examined the interaction between small worlds and technical competition pressure in Model 1.4. The results showed that their interaction (β = −2.629, *p* < 0.1) intensified the tendency against the radical innovation of enterprise, supporting Hypothesis 2a.

Hypothesis 2b predicted that being embedded in a small world would strengthen the effect of competitor-weighted centrality on a firm’s incremental innovation. Similarly, we observed that the coefficient of Smallworld (β = −0.340, *p* < 0.05) was negative and significant (Model 2.3), which means that enterprises being structured in the small world was also not beneficial for their innovation. Furthermore, we examined the interaction between firms in a small world and competitor-weighted centrality in Model 2.4. The results showed that their interaction (β = 2.897, *p* < 0.05) significantly enhanced the enterprises’ exploitative innovation, supporting Hypothesis 2b.

### 5.3. Robustness Test

To enhance the reliability of the results, we conducted three robustness tests. The results are presented in Table 4. First, the fixed-effects Poisson (FEP) panel regression was used as an alternative to the abovementioned models. Second, since the dependent variable contained many 0 values, we also conducted a robustness test with a zero-inflation Poisson (ZIP) regression. Finally, we changed the measurement window of the dependent variable. The 5-year moving window was replaced with 4 years. Radical and incremental patents were judged by whether the patent IPC code had occurred within the previous four years. The robustness test results once again proved the reliability of our results.

## 6. Conclusions and Implications

Based on the constructed technical competition network of wind energy enterprises, this study examined how two significant network structures (competitor-weighted centrality and embeddedness in small-world clusters) affect network firms’ ambidexterity of green technology innovation from a social embeddedness perspective. Our results verified our hypotheses. (1) Competitor-weighted centrality contributed to an enterprise’s incremental and radical green innovation. This is because stronger competitors put the focal firm under higher technical pressure, which motivates the firm to keep exploring and exploiting to improve its innovation. Moreover, powerful rivals from the network can provide the focal firm with more useful information that can stimulate its green innovation. (2) The interaction between competitor-weighted centrality and being embedded in small-world clusters can promote the focal firm’s incremental green innovation but can hinder its radical green innovation. Our findings elucidate the critical roles of competitors and the embedded structural features, providing enterprises with a benchmark to balance their incremental and radical innovative activities.

### 6.1. Theoretical Contributions

First, our research enhances the understanding of how competition affects innovation by exploring the ambidexterity of green innovation. Our investigation of innovation ambidexterity provides an integrated picture of how competitive relationships affect firm innovation. Firms embedded in small-world clusters face competitors that are more stable and less diverse [10], and the repeated interactions between those firms facilitate information transfer [17]. These factors make firms more sensitive to the dynamics of their rivals and, thus, take faster actions to respond. Therefore, we argue that stable competition promotes enterprise innovation, but only incremental innovation. From a long-term perspective, this stable competitive relationship would reinforce cognitive barriers and information insularity, which is not beneficial to a firm’s radical innovation.

Second, our study provides new insights into the relationship between competition structures and technological innovation management. We focus on two significant structures of a competition network. One is the competitor-weighted centrality of a firm in the network. Although previous studies have illuminated the importance of the centrality of a firm’s position in a collaboration network [11,18], there are some differences between cooperation networks and competition networks in terms of information exchange [14,15]. To the best of our knowledge, this is the first study that investigates the competitiveness of a focal firm’s competitors and how its competitor-weighted centrality affects the firm’s green technology innovation. Our findings suggest that the more central a firm is within a competition network, the higher its performance in green technology innovation. This is partly because a central competitor can impose higher competitive pressure on the focal firm and provide it with more valuable information, which instigates the focal firm to constantly search for and learn from its competitors. These findings support the argument from previous studies that competitors can be a valuable resource rather than a threat, as most previous studies have suggested [19].

Third, the findings related to a firm’s embeddedness in small-world clusters contribute to research on the social embeddedness perspective and green innovation literature. On the one hand, this paper complements previous studies on the social embeddedness perspective, which focuses their attention merely on other network structures [10,15,16,19]. Small-world clusters are important but often overlooked network structural features. We discovered that embeddedness in a small world is a suitable embodiment for understanding the competitive relationship between firms within a competition network. Therefore, this study enriches the research on the antecedent factors of green innovation. Similar to environmental policy, the small-world characteristic is an important dimension for measuring the external environment at the firm level. Our results show that firms embedded in different positions within a small-world cluster can receive different kinds of competitive pressure and obtain different technical information, which can lead the firms to employ different reactions and adopt different technological innovation strategies. These findings also support the idea that the environment in which a firm is embedded has a significant influence on its competitive capacity [16] and search strategies [52]. 

### 6.2. Managerial Implications

Our research has several important managerial implications for practitioners involved in green technology innovation. (1) Managers should treat their competitors as vital resources rather than threats to their technology innovation. Since competitive relationships can be an important channel for information flow between rivals, competitors with high technical capabilities can inspire the focal firm to engage in more innovation activities, and thus, improve their performance through such information flows and identify promising technological directions. (2) Managers can leverage competitive pressure to enhance their innovation performance. Managers need to be aware that an appropriate level of competitive pressure can become a driving force for firms to improve their abilities in innovation. (3) A familiar competition environment can be conducive to incremental innovation. Stable and repeated linkages in a small world can provide regular information and share technical improvements on slim trajectories, which can help firms perceive the insignificant dynamics of the competitors and respond to those changes quickly, thus improving incremental green innovation. (4) Finding new competitors is an efficient way to achieve radical green innovation. Enterprises should appropriately extend their sights to competitors outside their clique, such as firms in different markets or utilizing different technologies. These competitors can provide fresh ideas to stimulate the firm’s explorative capabilities and contribute to its radical green innovation. 

### 6.3. Limitations and Future Directions

This study has some limitations. First, the measurement of green technology innovation outcomes can be more diverse. Although patents are an important indicator of technological innovativeness, other measurements, such as tacit knowledge, technology or trade secrets, and technical standards, can also be used to measure technology innovation outcomes. Second, this study selected only the top 120 leading enterprises as the empirical sample. Future research may apply more advanced data pre-processing and analysis methods to increase the sample size. For example, methods such as natural language processing and big data analytics can be used as efficient complementary methods in data collection and text pre-processing [78,79]. Finally, future studies can explore the features of the technology convergence network mentioned in Section 4.2. This study only investigated the competition network constructed based on the collected patent data, whereas the technology convergence network that laid the foundations for a firm’s competition has not been explored. The interactions of the two networks may provide new insight into the competitive behaviors of the focal firms.

## Figures and Tables

**Table 1:** Summary of variables.

Variables	Measures	Literature Support	Data Source
Radical green innovation (RGI)	The number of patents that contain new technical categories. The new technology category refers to the technical class that a firm has never used in patents filed in the past five years.	[41,64,73,74]	Derwent Innovation database
Incremental green innovation (IGI)	The number of patents that contain only existing technical categories. The existing technical category refers to a familiar technical class (which has appeared at least once) in the past five years.		
Competitor-weighted centrality (CWC)	Eigenvector centrality	[11,16,69]	Firm competition network
Small-world clusters (Smallworld)	The degree to which the focal firm clusters with neighboring firms into a group (the average clustering coefficient)	[12,71,72]	Firm competition network
Firm size	The number of employees (thousands)	[12,75]	Standard and Poor’s Compustat Xpressfeed data; Compustat North America/Global database; Corporate Financial annual report; Company websites;EBSCOhost company information
Cash flow from operating activities (CFOA)	The annual cash flow of operating activities of firms (million dollars (log)).	[2,67]	
R&D expenditure	The annual investment in firms’ research and development activities (million dollars (log)).	[75]	
Firm age	The number of years since the established date	[12]	
Firm’s technology elements	The total number of IPC classes contained in firm patents	[16,76]	Derwent Innovation database

**Table 2:** Descriptive statistics and correlations.

	Mean	SD	RGI	IGI	CWC	Smallworld	Size	lnCFOA	lnRD	Age
RGI	2.747	4.668								
IGI	3.711	11.33	0.828 ***							
CWC	0.0598	0.115	0.917 ***	0.956 ***						
Smallworld	1.745	1.177	−0.259 ***	−0.213 ***	−0.285 ***					
Size	78.62	116.7	0.128 ***	0.156 ***	0.144 ***	−0.03				
lnCFOA	53.02	49.12	0.029	0.055	0.029	−0.017	0.243 ***			
lnRD	10.09	15.03	0.022	0.038	0.015	−0.052	0.284 ***	0.559 ***		
Age	6.910	2.907	0.078 *	0.102 **	0.100 **	0.007	0.445 ***	0.058	0.113 **	
Tcs	5.943	2.576	0.936 ***	0.879 ***	0.937 ***	−0.279 ***	0.138 ***	0.055	0.028	0.075 *

*** *p* < 0.01, ** *p* < 0.05, * *p* < 0.1.

**Table 3:** Results of the fixed-effects negative binomial panel analysis.

VARIABLES	Radical Green Innovation				Incremental Green Innovation			
	Model 1.1	Model 1.2	Model 1.3	Model 1.4	Model 2.1	Model 2.2	Model 2.3	Model 2.4
CWC		2.344 ***	2.014 **	3.722 ***		4.785 ***	4.273 ***	2.744 ***
Smallworld			−0.219 ***	−0.191 **			−0.340 **	−0.456 ***
CWC * Smallworld				−2.629 *				2.897 **
Size	−0.003 *	−0.003	−0.003	−0.003	0.004 **	0.001	0.001	0.002
lnCFOA	−0.026	−0.019	−0.022	−0.018	0.013	0.068 *	0.054	0.051
lnRD	0.029	−0.013	−0.01	−0.014	0.024	−0.064	−0.048	−0.05
Age	−0.057 ***	−0.037 **	−0.028	−0.022	−0.015 ***	−0.001	0.003	0.005
Tcs	0.030 ***	0.020 ***	0.020 ***	0.026 ***	0.011 ***	−0.008 **	−0.006 *	−0.014 ***
Constant	14.881	12.077	8.896	8.171	1.734 ***	1.096 *	1.530 **	1.757 ***
Observations	252	252	252	252	207	207	207	207
Number of firms	53	53	53	53	38	38	38	38
Firm fixed effects	Include	Include	Include	Include	Include	Include	Include	Include
Wald chi-square	156.5	158.97	165.89	169.57	27.88	98.35	102.28	111.53
Log-likelihood	−301.3	−296.8	−291.1	−289.4	−266	−250.3	−246.4	−244.1

*** *p* < 0.01, ** *p* < 0.05, * *p* < 0.1.

**Table 4:** Robustness test.

VARIABLES	Radical Innovation			Incremental Innovation		
	FEP	ZIP	4-Year	FEP	ZIP	4-Year
CWC	3.732 ***	2.972 ***	3.901 ***	3.318 ***	3.024 ***	2.684 ***
Smallworld	−0.191 **	−0.415 ***	−0.183 **	−0.678 ***	−1.487 ***	−0.527 ***
CWC * Smallworld	−2.626 *	−2.942 **	−2.264 *	2.470 ***	4.130 ***	2.955 **
Size	−0.003	−0.001 *	−0.003	0.002 *	−0.001 *	0.002
lnCFOA	−0.018	0.006	−0.02	0.078 ***	0.123 ***	0.051
lnRD	−0.014	−0.019	−0.016	−0.069	−0.134 ***	−0.053
Age	−0.023	0.001	−0.013	0.043 ***	0.003 ***	0.006
Tcs	0.026 ***	0.028 ***	0.022 ***	−0.016 ***	−0.013 ***	−0.014 ***
Constant		1.221 ***	6.781		2.324 ***	1.714 **
Observations	252	259	252	207	259	199
Number of firms	53		53	38		36
Wald chi-square	175.5		169.77	169.29		109.86
LR chi		2657.99			2657.99	
Log-likelihood	−289.4	−443.6	−292.3	−244.8	−420.1	−239.3

*** *p* < 0.01, ** *p* < 0.05, * *p* < 0.1.

## Data Availability

The data that support the findings of this study are available from the corresponding author upon reasonable request.
